# (*E*)-3-Bromo-*N*′-(4-hydr­oxy-3-nitro­benzyl­idene)benzohydrazide

**DOI:** 10.1107/S1600536809024131

**Published:** 2009-07-01

**Authors:** Guo-Biao Cao, Xiao-Ya Wang

**Affiliations:** a Department of Chemistry, Ankang University, Ankang Shanxi 725000, People’s Republic of China; b Department of Biology, Ankang University, Ankang Shanxi 725000, People’s Republic of China

## Abstract

The title compound, C_14_H_10_BrN_3_O_4_, was synthesized by the reaction of 4-hydr­oxy-3-nitro­benzaldehyde with an equimolar quantity of 3-bromo­benzohydrazide in methanol. The mol­ecule displays an *E* configuration about the C=N bond. The dihedral angle between the two benzene rings is 4.6 (2)°. The nitro group is almost coplanar with the attached benzene ring [dihedral angle = 4.7 (2)°]. In the crystal structure, mol­ecules are linked into sheets parallel to (100) by inter­molecular N—H⋯O, O—H⋯N and O—H⋯O hydrogen bonds.

## Related literature

For the crystal structures of hydrazone compounds, see: Mohd Lair *et al.* (2009 ▶); Fun *et al.* (2008 ▶); Li & Ban (2009 ▶); Zhu *et al.* (2009 ▶); Yang (2007 ▶); You *et al.* (2008 ▶). For hydrazone compounds reported previously by our group, see: Qu *et al.* (2008 ▶); Yang *et al.* (2008 ▶); Cao & Lu (2009*a* ▶,*b* ▶).
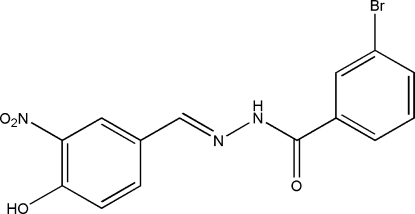


## Experimental

### 

#### Crystal data

C_14_H_10_BrN_3_O_4_
*M* *_r_* = 364.16
Monoclinic, 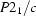

*a* = 12.323 (1) Å
*b* = 13.697 (1) Å
*c* = 8.430 (1) Å
β = 97.133 (2)°
*V* = 1411.9 (2) Å^3^
*Z* = 4
Mo *K*α radiation
μ = 2.93 mm^−1^
*T* = 298 K
0.23 × 0.21 × 0.20 mm

#### Data collection

Bruker SMART CCD area-detector diffractometer
Absorption correction: multi-scan (*SADABS*; Bruker, 2001 ▶) *T*_min_ = 0.552, *T*_max_ = 0.592 (expected range = 0.519–0.556)
8326 measured reflections
2946 independent reflections
1834 reflections with *I* > 2σ(*I*)
*R*_int_ = 0.036

#### Refinement

*R*[*F* ^2^ > 2σ(*F* ^2^)] = 0.048
*wR*(*F* ^2^) = 0.128
*S* = 1.04
2946 reflections
203 parameters
1 restraint
H atoms treated by a mixture of independent and constrained refinement
Δρ_max_ = 0.66 e Å^−3^
Δρ_min_ = −0.76 e Å^−3^

### 

Data collection: *SMART* (Bruker, 2007 ▶); cell refinement: *SAINT* (Bruker, 2007 ▶); data reduction: *SAINT*; program(s) used to solve structure: *SHELXTL* (Sheldrick, 2008 ▶); program(s) used to refine structure: *SHELXTL*; molecular graphics: *SHELXTL*; software used to prepare material for publication: *SHELXTL*.

## Supplementary Material

Crystal structure: contains datablocks global, I. DOI: 10.1107/S1600536809024131/ci2834sup1.cif

Structure factors: contains datablocks I. DOI: 10.1107/S1600536809024131/ci2834Isup2.hkl

Additional supplementary materials:  crystallographic information; 3D view; checkCIF report

## Figures and Tables

**Table 1:** Hydrogen-bond geometry (Å, °)

*D*—H⋯*A*	*D*—H	H⋯*A*	*D*⋯*A*	*D*—H⋯*A*
N2—H2⋯O4^i^	0.90 (1)	2.06 (2)	2.914 (4)	159 (4)
O3—H3⋯N1^ii^	0.82	2.56	2.999 (4)	115
O3—H3⋯O4^ii^	0.82	2.30	2.992 (4)	142

Symmetry codes: (i) 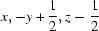; (ii) 
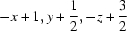
.

**Fig. 1.**
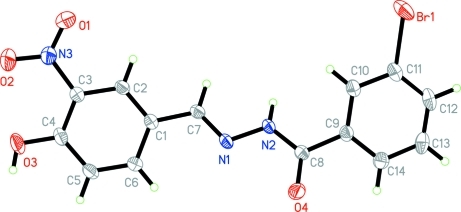
The molecular structure of the title compound, showing 30% displacement ellipsoids.

**Fig. 2.**
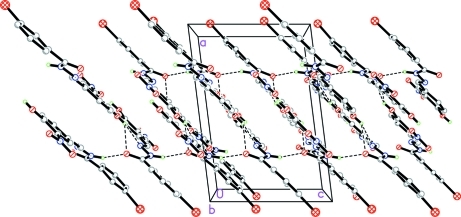
The molecular packing of the title compound, viewed along the b axis. H atoms not involved in hydrogen bonding (dashed lines) have been omitted.

## supplementary crystallographic information

### Comment

Study on the crystal structures of hydrazone derivatives is a hot topic in structural chemistry. In the last few years, the crystal structures of a large number of hydrazone compounds have been reported (Mohd Lair *et al.*, 2009; Fun *et al.*, 2008; Li & Ban, 2009; Zhu *et al.*, 2009; Yang, 2007; You *et al.*, 2008). As a continuation of our work in this area (Qu *et al.*, 2008; Yang *et al.*, 2008; Cao & Lu, 2009a,b), the title new hydrazone compound derived from the reaction of 4-hydroxy-3-nitrobenzaldehyde with an equimolar quantity of 3-bromobenzohydrazide is reported.

In the title compound (Fig. 1), the dihedral angle between the two benzene rings is 4.6 (2)°. The molecule displays an *E* configuration about the C═N bond. In the crystal structure, molecules are linked through intermolecular N—H···O, O—H···N, and O—H···O hydrogen bonds (Table 1) to form layers parallel to the (100) (Fig. 2).

### Experimental

The title compound was prepared by refluxing equimolar quantities of 4-hydroxy-3-nitrobenzaldehyde with 3-bromobenzohydrazide in methanol. Colourless block-like crystals were formed by slow evaporation of the solution in air.

### Refinement

Atom H2 was located in a difference Fourier map and refined isotropically, with the N-H distance restrained to 0.90 (1) Å. The other H atoms were placed in idealized positions and constrained to ride on their parent atoms, with C-H distances of 0.93 Å, O-H distance of 0.82 Å, and with *U*_iso_(H) set at 1.2*U*_eq_(C) and 1.5*U*_eq_(O). A rotating group model was used for the OH group.

### Crystal data

**Table:**

C_14_H_10_BrN_3_O_4_	*F*(000) = 728
*M**_r_* = 364.16	*D*_x_ = 1.713 Mg m^−^^3^
Monoclinic, *P*2_1_/*c*	Mo *K*α radiation, λ = 0.71073 Å
Hall symbol: -P 2ybc	Cell parameters from 1828 reflections
*a* = 12.323 (1) Å	θ = 2.7–24.5°
*b* = 13.697 (1) Å	µ = 2.93 mm^−^^1^
*c* = 8.430 (1) Å	*T* = 298 K
β = 97.133 (2)°	Block, colourless
*V* = 1411.9 (2) Å^3^	0.23 × 0.21 × 0.20 mm
*Z* = 4	

### Data collection

**Table:**

Bruker SMART CCD area-detector diffractometer	2946 independent reflections
Radiation source: fine-focus sealed tube	1834 reflections with *I* > 2σ(*I*)
graphite	*R*_int_ = 0.036
ω scans	θ_max_ = 26.6°, θ_min_ = 1.7°
Absorption correction: multi-scan (*SADABS*; Bruker, 2001)	*h* = −14→15
*T*_min_ = 0.552, *T*_max_ = 0.592	*k* = −16→17
8326 measured reflections	*l* = −10→10

### Refinement

**Table:**

Refinement on *F*^2^	Primary atom site location: structure-invariant direct methods
Least-squares matrix: full	Secondary atom site location: difference Fourier map
*R*[*F*^2^ > 2σ(*F*^2^)] = 0.048	Hydrogen site location: inferred from neighbouring sites
*wR*(*F*^2^) = 0.128	H atoms treated by a mixture of independent and constrained refinement
*S* = 1.04	*w* = 1/[σ^2^(*F*_o_^2^) + (0.0583*P*)^2^ + 0.7063*P*] where *P* = (*F*_o_^2^ + 2*F*_c_^2^)/3
2946 reflections	(Δ/σ)_max_ = 0.001
203 parameters	Δρ_max_ = 0.66 e Å^−^^3^
1 restraint	Δρ_min_ = −0.76 e Å^−^^3^

### Special details

**Table:**

Geometry. All esds (except the esd in the dihedral angle between two l.s. planes) are estimated using the full covariance matrix. The cell esds are taken into account individually in the estimation of esds in distances, angles and torsion angles; correlations between esds in cell parameters are only used when they are defined by crystal symmetry. An approximate (isotropic) treatment of cell esds is used for estimating esds involving l.s. planes.
Refinement. Refinement of F^2^ against ALL reflections. The weighted R-factor wR and goodness of fit S are based on F^2^, conventional R-factors R are based on F, with F set to zero for negative F^2^. The threshold expression of F^2^ > 2sigma(F^2^) is used only for calculating R-factors(gt) etc. and is not relevant to the choice of reflections for refinement. R-factors based on F^2^ are statistically about twice as large as those based on F, and R- factors based on ALL data will be even larger.

### Fractional atomic coordinates and isotropic or equivalent isotropic displacement parameters (Å^2^)

**Table:**

	*x*	*y*	*z*	*U*_iso_*/*U*_eq_	
Br1	1.07300 (4)	0.14793 (4)	0.14031 (6)	0.0779 (2)	
N1	0.6951 (2)	0.3333 (2)	0.5669 (3)	0.0418 (7)	
N2	0.7511 (2)	0.2683 (2)	0.4801 (3)	0.0402 (7)	
N3	0.6518 (3)	0.7702 (2)	0.5895 (4)	0.0525 (8)	
O1	0.7225 (3)	0.7781 (2)	0.5036 (4)	0.0735 (9)	
O2	0.6110 (4)	0.8411 (2)	0.6463 (5)	0.0963 (13)	
O3	0.4820 (2)	0.7296 (2)	0.7858 (4)	0.0660 (8)	
H3	0.4390	0.7082	0.8440	0.099*	
O4	0.7360 (3)	0.14917 (19)	0.6595 (3)	0.0731 (10)	
C1	0.6432 (3)	0.4996 (2)	0.5965 (4)	0.0356 (8)	
C2	0.6664 (3)	0.5944 (2)	0.5594 (4)	0.0366 (8)	
H2A	0.7163	0.6068	0.4878	0.044*	
C3	0.6159 (3)	0.6722 (3)	0.6279 (4)	0.0389 (8)	
C4	0.5363 (3)	0.6554 (3)	0.7283 (4)	0.0444 (9)	
C5	0.5151 (3)	0.5582 (3)	0.7673 (4)	0.0471 (9)	
H5	0.4645	0.5452	0.8378	0.057*	
C6	0.5678 (3)	0.4816 (3)	0.7031 (4)	0.0431 (9)	
H6	0.5530	0.4177	0.7311	0.052*	
C7	0.6986 (3)	0.4217 (2)	0.5201 (4)	0.0390 (8)	
H7	0.7374	0.4368	0.4354	0.047*	
C8	0.7693 (3)	0.1783 (3)	0.5364 (4)	0.0415 (9)	
C9	0.8344 (3)	0.1142 (2)	0.4401 (4)	0.0373 (8)	
C10	0.9097 (3)	0.1540 (3)	0.3480 (4)	0.0412 (9)	
H10	0.9198	0.2212	0.3441	0.049*	
C11	0.9692 (3)	0.0920 (3)	0.2625 (4)	0.0475 (9)	
C12	0.9556 (3)	−0.0073 (3)	0.2673 (5)	0.0562 (11)	
H12	0.9957	−0.0482	0.2084	0.067*	
C13	0.8825 (3)	−0.0454 (3)	0.3592 (5)	0.0541 (11)	
H13	0.8734	−0.1127	0.3633	0.065*	
C14	0.8215 (3)	0.0146 (3)	0.4469 (4)	0.0442 (9)	
H14	0.7723	−0.0123	0.5098	0.053*	
H2	0.762 (4)	0.284 (3)	0.380 (2)	0.080*	

### Atomic displacement parameters (Å^2^)

**Table:**

	*U*^11^	*U*^22^	*U*^33^	*U*^12^	*U*^13^	*U*^23^
Br1	0.0654 (4)	0.0956 (4)	0.0811 (4)	0.0124 (3)	0.0426 (3)	−0.0031 (3)
N1	0.0517 (19)	0.0410 (18)	0.0368 (16)	0.0099 (14)	0.0213 (15)	0.0001 (13)
N2	0.0514 (18)	0.0363 (16)	0.0370 (17)	0.0089 (14)	0.0211 (15)	−0.0005 (13)
N3	0.063 (2)	0.0412 (19)	0.055 (2)	0.0061 (17)	0.0153 (18)	−0.0007 (15)
O1	0.079 (2)	0.0502 (18)	0.098 (2)	−0.0069 (15)	0.036 (2)	0.0067 (15)
O2	0.142 (3)	0.0425 (19)	0.117 (3)	0.0176 (19)	0.066 (3)	−0.0030 (17)
O3	0.0595 (19)	0.0610 (18)	0.084 (2)	0.0089 (14)	0.0343 (16)	−0.0240 (16)
O4	0.136 (3)	0.0419 (16)	0.0512 (18)	0.0135 (16)	0.0520 (19)	0.0077 (12)
C1	0.0356 (19)	0.0390 (19)	0.0329 (19)	0.0037 (15)	0.0067 (16)	−0.0029 (14)
C2	0.038 (2)	0.040 (2)	0.0325 (18)	0.0033 (16)	0.0098 (16)	0.0017 (15)
C3	0.040 (2)	0.039 (2)	0.039 (2)	0.0015 (15)	0.0091 (16)	−0.0020 (15)
C4	0.040 (2)	0.048 (2)	0.046 (2)	0.0051 (17)	0.0109 (18)	−0.0127 (17)
C5	0.046 (2)	0.053 (2)	0.046 (2)	−0.0032 (18)	0.0214 (18)	−0.0066 (17)
C6	0.047 (2)	0.041 (2)	0.044 (2)	−0.0028 (17)	0.0170 (18)	−0.0011 (16)
C7	0.044 (2)	0.042 (2)	0.0338 (19)	0.0043 (16)	0.0142 (16)	0.0030 (15)
C8	0.054 (2)	0.038 (2)	0.036 (2)	0.0040 (17)	0.0182 (18)	−0.0010 (15)
C9	0.043 (2)	0.0367 (19)	0.0326 (19)	0.0069 (15)	0.0054 (16)	−0.0014 (14)
C10	0.042 (2)	0.043 (2)	0.039 (2)	0.0085 (16)	0.0062 (17)	−0.0027 (16)
C11	0.044 (2)	0.055 (2)	0.045 (2)	0.0103 (18)	0.0102 (19)	−0.0068 (18)
C12	0.053 (3)	0.056 (3)	0.060 (3)	0.019 (2)	0.008 (2)	−0.021 (2)
C13	0.059 (3)	0.036 (2)	0.065 (3)	0.0088 (19)	−0.002 (2)	−0.0138 (18)
C14	0.046 (2)	0.042 (2)	0.044 (2)	0.0005 (17)	0.0044 (18)	−0.0029 (17)

### Geometric parameters (Å, °)

**Table:**

Br1—C11	1.900 (4)	C4—C5	1.404 (5)
N1—C7	1.276 (4)	C5—C6	1.380 (5)
N1—N2	1.389 (4)	C5—H5	0.93
N2—C8	1.329 (4)	C6—H6	0.93
N2—H2	0.898 (10)	C7—H7	0.93
N3—O1	1.206 (4)	C8—C9	1.495 (5)
N3—O2	1.219 (4)	C9—C14	1.376 (5)
N3—C3	1.462 (5)	C9—C10	1.392 (5)
O3—C4	1.341 (4)	C10—C11	1.381 (5)
O3—H3	0.82	C10—H10	0.93
O4—C8	1.229 (4)	C11—C12	1.373 (6)
C1—C2	1.374 (5)	C12—C13	1.362 (6)
C1—C6	1.393 (5)	C12—H12	0.93
C1—C7	1.459 (5)	C13—C14	1.388 (5)
C2—C3	1.394 (5)	C13—H13	0.93
C2—H2A	0.93	C14—H14	0.93
C3—C4	1.392 (5)		
			
C7—N1—N2	114.0 (3)	C1—C6—H6	119.9
C8—N2—N1	118.6 (3)	N1—C7—C1	121.5 (3)
C8—N2—H2	121 (3)	N1—C7—H7	119.3
N1—N2—H2	119 (3)	C1—C7—H7	119.3
O1—N3—O2	121.9 (4)	O4—C8—N2	122.9 (3)
O1—N3—C3	118.5 (3)	O4—C8—C9	121.7 (3)
O2—N3—C3	119.6 (3)	N2—C8—C9	115.4 (3)
C4—O3—H3	109.5	C14—C9—C10	120.0 (3)
C2—C1—C6	119.2 (3)	C14—C9—C8	119.2 (3)
C2—C1—C7	118.0 (3)	C10—C9—C8	120.9 (3)
C6—C1—C7	122.8 (3)	C11—C10—C9	119.0 (3)
C1—C2—C3	120.8 (3)	C11—C10—H10	120.5
C1—C2—H2A	119.6	C9—C10—H10	120.5
C3—C2—H2A	119.6	C12—C11—C10	121.3 (4)
C4—C3—C2	120.7 (3)	C12—C11—Br1	120.5 (3)
C4—C3—N3	122.8 (3)	C10—C11—Br1	118.2 (3)
C2—C3—N3	116.6 (3)	C13—C12—C11	119.2 (4)
O3—C4—C3	121.1 (3)	C13—C12—H12	120.4
O3—C4—C5	121.2 (3)	C11—C12—H12	120.4
C3—C4—C5	117.7 (3)	C12—C13—C14	121.0 (4)
C6—C5—C4	121.3 (3)	C12—C13—H13	119.5
C6—C5—H5	119.4	C14—C13—H13	119.5
C4—C5—H5	119.4	C9—C14—C13	119.5 (4)
C5—C6—C1	120.2 (3)	C9—C14—H14	120.2
C5—C6—H6	119.9	C13—C14—H14	120.2

### Hydrogen-bond geometry (Å, °)

**Table:**

*D*—H···*A*	*D*—H	H···*A*	*D*···*A*	*D*—H···*A*
N2—H2···O4^i^	0.90 (1)	2.06 (2)	2.914 (4)	159 (4)
O3—H3···N1^ii^	0.82	2.56	2.999 (4)	115
O3—H3···O4^ii^	0.82	2.30	2.992 (4)	142

Symmetry codes: (i) *x*, −*y*+1/2, *z*−1/2; (ii) −*x*+1, *y*+1/2, −*z*+3/2.
